# Association of overweight/obesity and digestive system cancers: A meta-analysis and trial sequential analysis of prospective cohort studies

**DOI:** 10.1371/journal.pone.0318256

**Published:** 2025-04-01

**Authors:** Ji Ren, Chunyan Tang, Jinghe Wang, Yanan Wang, Dongying Yang, Jianming Sheng, Shili Zhu, Yunli Liu, Xiaoqi Li, Wei Liu

**Affiliations:** 1 Department of Medicine and Health, Dezhou University, Dezhou, China; 2 Department of Nursing, Dezhou Municipal Hospital (Dezhou University Affiliated Hospital), Dezhou, China; Debre Tabor University, ETHIOPIA

## Abstract

**Background:**

Previous researches have reported correlations between overweight/obesity and common digestive system cancers (DSCs), including gastric, liver, esophageal, colorectal, and pancreatic cancers. However, the inconsistency in defining overweight/obesity and the risk of recall bias from case-control and retrospective cohort studies may influence existing results. Therefore, we aimed to validate the relationship between overweight/obesity and common DSCs by combining prospective cohort studies based on the World Health Organization (WHO) criteria for defining overweight/obesity.

**Methods:**

A comprehensive literature search was conducted across PubMed, Embase, Web of Science, and Cochrane databases, covering all publications up to February 7, 2024. The inclusion criteria focused on prospective cohort studies that examined the link between overweight/obesity and risks of DSCs. R software 4.1.3 and STATA 12 were utilised to calculate the relative risk (RR), with 95% confidence interval (CI) and prediction interval (PI). TSA v0.9.5.10 Beta software was used for trial sequential analysis (TSA).

**Results:**

The meta-analysis encompassed 39 articles. The overall analysis showed that compared with normal weight, overweight/obesity increased the risks of liver cancer (overweight: RR [95% CI] =  1.237 [1.112-1.377]; 95% PI: 0.888-1.725; obesity: RR [95% CI] =  1.642 [1.466-1.839]; 95% PI: 1.143-2.358) and colorectal cancer (overweight: RR [95% CI] =  1.124 [1.056-1.197]; 95% PI: 0.931-1.357; obesity: RR [95% CI] =  1.366 [1.242-1.503]; 95% PI: 0.959-1.945) in the total population. Subgroup analysis revealed that overweight (RR [95% CI] =  1.237 [1.165-1.314]; 95% PI: 1.154-1.327) and obesity (RR [95% CI] =  1.306 [1.152-1.480]; 95% PI: 1.108-1.539) were associated with an increased risk of pancreatic cancer only in women, and overweight also increased the gastric cancer risk of women (RR [95% CI] =  1.041 [1.013-1.070], 95% PI: 0.806-1.230). No significant association of overweight/obesity and esophageal cancer was observed in both male and female.

**Conclusion:**

Our study suggested that overweight/obesity elevated the risks of liver and colorectal cancer in both men and women. No significant association was found between overweight/obesity and the risk of developing esophageal cancer. Clinicians are advised to consider weight control as an effective measure for preventing pancreatic, liver, and colorectal cancers.

## 1. Introduction

Globally, digestive system cancers (DSCs) constitute about 30% of all cancer cases and are the leading cause of cancer-related deaths worldwide, presenting a significant global health challenge [1–3]. DSC, which mainly includes gastric, esophageal, liver, pancreatic, and colorectal cancers, has emerged as the primary cause of mortality globally [4]. Colon and rectum, gastric, liver and esophageal cancers, are ranked within the top 5 diagnoses in China, which account for 41% of new cancer cases and 49% of mortality [5]. Based on 2020 cancer data, cancers of the colorectum, stomach, liver, and esophagus rank within the global top 10 most prevalent cancers. The year saw 604,000 new instances of esophageal cancer, over 1.0 million gastric cancer cases, 906,000 newly diagnosed liver cancer patients, and 1.9 million individuals were reported with new cases of colorectal cancer [5]. In recent years, the incidence of being overweight and obese has surged, now representing over a third of the global populace [6]. These conditions are typically classified based on body mass index (BMI), with overweight defined as a BMI of ≥  25 to <  30 kg/m^2^ and obesity as a BMI of ≥  30 kg/m^2^ [7]. Such adiposity-related conditions are fundamental contributors to a myriad of health issues, including hypertension, diabetes mellitus, cardiovascular disorders, pancreatitis, gallbladder disease, osteoarthritis, and sleep apnea [8].

Due to obesity being the fastest growing disease in the world [9,10], the evidences and mechanisms of gastrointestinal cancers caused by obesity have been suggested [11,12]. More and more evidences suggest that obesity elevates the likelihood of developing certain types of cancer, including pancreatic, colorectal, liver, gastric, and esophageal cancers [13–17]. Earlier research has indicated that being overweight or obese significantly increases the risk of developing DSCs when compared to maintaining a normal weight [18–20]. Data from case-control and cohort analyses have pointed to a link between obesity and a heightened risk of developing gastric cancer relative to those with a normal weight status [21]. Moreover, a comprehensive meta-analysis that consolidated findings from both cohort and case-control research demonstrated a correlation between excess weight and an increased incidence of early-onset colorectal cancer relative to those of normal weight [22]. Additional analysis derived from 25 observational studies highlighted that overweight and obesity were linked to an increased likelihood of esophageal adenocarcinoma (EADC) development, yet interestingly, these conditions appeared to confer a protective effect against the development of esophageal squamous cell carcinoma (ESCC) [23]. The World Cancer Research Fund (WCRF) advises that individuals diagnosed with cancer should maintain their BMI within the normal range.

Nonetheless, research highlighting the connection between overweight/obesity and DSC could be influenced by potential biases, due to their reliance on retrospective cohort or case-control study designs. Moreover, studies had different definitions of overweight/obesity, leading to an undetermined relationship between overweight/obesity and common DSCs. Hence, this study, based on the World Health Organization (WHO) criteria for overweight and obesity, was carried out to examine prospective cohorts to ascertain the association between overweight/obesity and DSCs through a meta-analysis.

## 2. Methods

This meta-analysis was enrolled in the International Prospective Register of Systematic Reviews (registration number CRD42023413565). The Preferred Reporting Items for Systematic Reviews and Meta-Analyses (PRISMA) guideline was used and performed for this study [24].

### 2.1 Literature search

The authors systematically searched PubMed, Embase, Web of Science and Cochrane until April 2023 using the following MeSH terms and keywords: (“BMI”, “body mass index”, “obese”, “obesity”, “overweight”, “adiposity”) AND (“digestive system cancer”, “liver cancer”, “hepatic neoplasm”, “hepatocellular cancer”, “gastric cancer”, “stomach neoplasm”, “esophageal neoplasm”, “esophagus cancer”, “colorectal carcinoma”, “colorectal neoplasm”, “colorectal cancer”, “pancreas cancer”, “pancreatic cancer”, “pancreatic neoplasm”) AND (“concurrent study”, “cohort study”, “incidence study”). We updated literature search in all databases on February 7, 2024. The complete search strategy can be found in S1 File. The authors also searched the Chinese databases and reviewed references in relevant Chinese articles.

### 2.2 Study selection

The inclusion criteria were: (i) prospective cohort study; (ii) BMI was divided into four classifications: obesity (BMI ≥  30.0 kg/m^2^), overweight (BMI =  25.0 to 29.9 kg/m^2^), normal weight (BMI =  18.5 to 24.9 kg/m^2^), and underweight (BMI <  18.5 kg/m^2^) according to the WHO criteria [25]; (iii) providing relative risk (RR) or hazard ratio (HR) with 95% confidence interval (CI); (iv) the reference group was “normal weight” and the exposure groups were “overweight” or “obesity”. Exclusion criteria were: (i) case-control or retrospective cohort study; (ii) the reference group was “underweight”; (iii) only reporting the correlation between overweight/obesity and the incidence of rectal, colon cancer, pancreatic ductal adenocarcinoma or EADC; (iv) literature reviews, case reports, conference abstracts, and study protocols.

### 2.3 Data collection and quality assessment

Two authors independently extracted data using a standardised collection Excel file. From every research reviewed, we extracted pertinent details including the lead author’s name along with the publication year, the locale of the study, study period, population selection, age range of the subjects, the total number of participants and cancer cases, duration of follow-up, cancer varieties, and adjustments. The quality of cohort studies was assessed using the Newcastle-Ottawa Scale (NOS), which is divided into three sections [26]. Evaluations were conducted on a nine-point scale, categorizing studies into three levels of quality: those scoring 0-3 were classified as low quality; a rating of 4-6 indicated moderate quality; and studies achieving a score of 7-9 were recognized as high quality [27].

### 2.4 Statistical analysis

The association of overweight/obesity and the risks of DSCs are quantified through the HR and RR with 95% CI and prediction interval (PI). Because DSCs are rare in the general population, HR was directly considered as RR [28]. The Cochran’s Q and I^2^ statistics was used to assess the overall study heterogeneity [29,30]. A *P* value of 0.10 or lower, or an I^2^ greater than 50%, signals substantial heterogeneity among the studies, necessitating the use of a random-effects model; in contrast, when these criteria are not met, a fixed-effects model is chosen [31]. Through the application of the leave-one-out technique, we executed a sensitivity analysis to pinpoint potential heterogeneity origins among the studies. We visually evaluated publication bias through funnel plots and applied the trim-and-fill method for quantitative adjustment [32]. R software 4.1.3 and STATA 12 (StataCorp, College Station, TX, USA) were used to conduct the meta-analysis by the authors. All *P* <  0.05 indicated statistical significance.

### 2.5 Trial sequential analysis

We performed trial sequential analysis (TSA) using TSA v0.9.5.10 (www.ctu.dk/tsa). TSA can calculate the required information size (RIS) and whether more studies are needed [33]. In traditional meta-analyses, the frequent execution of significance tests on pooled data heightens the likelihood of type I errors. TSA, however, offers a methodological advantage by lowering this risk and fine-tuning the 95% CI of the RR. The execution of TSA entailed the establishment of O’ Brien-Fleming α-spending boundaries, which were determined using a 5% type I error and an 80% power.

## 3. Results

### 3.1 Literature search

**Fig 1:**
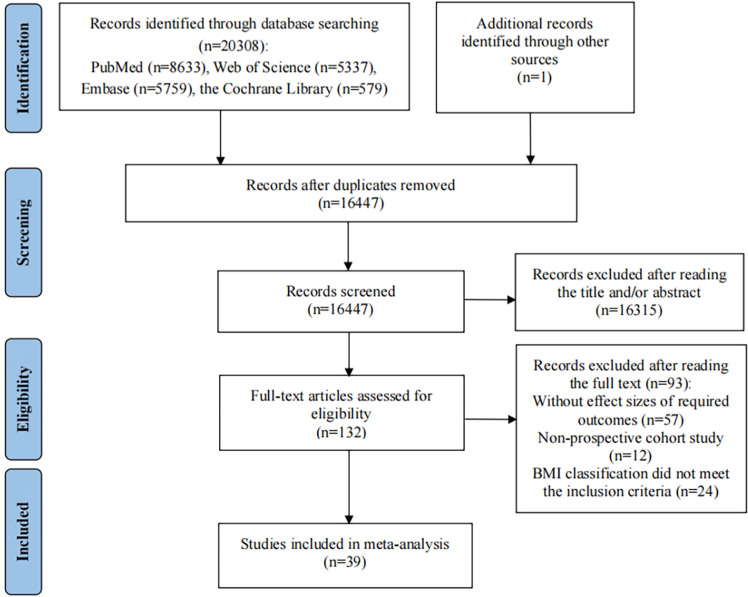
Flow diagram of the studies included in the meta-analysis.

The selection process was described in Fig 1. During the initial search, we obtained a total of 20,308 records, with 1 additional record from the Chinese databases. 3,861 literature were removed for duplicates, the remaining 16,447 literature were screened. After the titles and abstracts were screened against predefined inclusion criteria, 16,315 articles were excluded because of their irrelevant content. The remaining 132 articles were selected for full-text filtering, and 93 articles were excluded: 57 studies did not provide effect sizes of target cancers; 12 studies were not prospective cohort studies; 24 articles reported the BMI classification which did not meet the inclusion criteria. Ultimately, a total of 39 articles were included in the meta-analysis [34–72].

### 3.2 Characteristics and quality assessment of the included studies

All studies included in the meta-analysis have their characteristics detailed in Table 1. The 39 studies incorporated were all prospective cohort studies. 4 studies presented combined findings, incorporating pooled analyses from 7 prospective cohorts in the USA, Finland, and China, 14 in the USA, 10 in Japan, and 8 more also conducted in Japan. 4 studies explored the association between overweight/obesity and DSCs in men, while 6 investigations addressed this relationship in women. The quality assessment of the studies included was detailed in S2 File, with all studies evaluated to be of high quality.

**Table 1:** Characteristics of the studies included in the meta-analysis.

First author (year)	Region	Study period	Population selection	Age (range or mean age, years)	Participants	Cases	Follow-up time	Type of cancer	Adjustments
Shyam (2021)	England	1995-2014	The UK women’s cohort study	35-69	35,364 W	136 W	19.3 (median year)	1	Age, smoking, education and physical activity level
Oh (2005)	Korea	1992-2001.12	Members of the Korea National Health Insurance Corporation	≥ 20	781,283 M	9,106 M	10 years	1, 2, 3	Age
Koyanagi (2023)	Japan	1983-2014	10 population-based cohort studies in Japan	35-104	180,983 M + 213,264 W	9,033 M + 3631 W	13.8 (mean year)	2, 5	Sex, age, area, pack-years, alcohol consumption, and history of diabetes.
Liu (2016)	China	1996-2013.12	The Shanghai Women’s Health Study	40-70	68,253 W	1,608 W	15.1 (median year)	1, 2, 3, 4	Education, total energy intake, total vegetable and fruit intake, total meat intake, leisure-time physical activity, alcohol consumption, menopausal status, spouse smoking exposure, parity, family history of cancer, and waist-hip ratio
da Silva (2018)	Norway	1991-2014.12	The Norwegian Women and Cancer study	30-70	135,708 W	2,137 W	16.9 (mean year)	1, 4	Age, education and smoking status
Kuriyama (2005)	Japan	1984.1-1992.12	All residents 40 years of age or older in 3 municipalities of Miyagi Prefecture	≥ 40	12,485 M + 15,054 W	357 M + 152 W	9	2, 4	Age, smoking status, alcohol drinking status, consumption of meat, fish, fruits, green or yellow vegetables and bean-paste soup, type of health insurance (for women: menopausal status, parity, age at menarche, age at end of first pregnancy)
Liu (2019)	USA	1989-2011.12	The Nurses’ Health Study II	25-42	85,256 W	114 W	13.9 (median year)	4	Height, history of diabetes, smoking pack-years, physical activity, alcohol intake, regular use of aspirin, nonsteroidal anti-inflammatory drug use, multivitamin use, menopausal status, menopausal hormone use, and dietary intake
Engeland (2004)	Norway	1963-2002.12	General Norwegian population	20-74	963,653 M + 1,037,964 W	1,597 M + 648 W	23 (mean year)	5	Age at measurement and birth cohort
Zheng (2018)	USA	NA	The Prostate, Lung, Colorectal, and Ovarian (PLCO) Cancer Screening Trial	49-78	70,344 M + 68,885 W	1,196 M + 835 W	13	4	Randomization arm, sex, study center, race, family history of colorectal cancer and cigarette smoking status
Hagström (2018)	Sweden	1970-2012.12	Swedish men who underwent conscription into military service between 1969 and 1996	17-19	1,220,261 M	251 M	28.5 (mean year)	3	Age, year of birth, location of conscription, own and parental education, parental socioeconomic status, scores on intelligence test, cardiovascular capacity and muscular strength tests, systolic and diastolic blood pressures
Jee (2008)	Korea	1992-2006	Koreans who participated in 1 biennial National Health Insurance Corporation medical evaluation	30-95	770,556 M + 443,273 W	28,964 M + 7,823 W	10.8 (mean year)	1, 2, 3, 5	Age and smoking status
Samanic (2006)	Sweden	1971-1999.12	The Swedish Health Examination Database	34.3 (mean age)	362,552 M	1,255 M	19 (mean year)	1, 2, 5	Attained age and calendar year, smoking status, and relative to normal weight subjects
Lim (2022)	Korea	2009-2017	Individuals who underwent health check-up provided by the NHIC	≥ 20	2,757,017 M + W	13,441 M + W	6.78 (median year)	2	Age, sex, smoking, alcohol consumption, regular exercise, income, diabetes, hypertension, and dyslipidemia
Hoyt (2022)	USA	1993-2009	The Prostate, Lung, Colorectal, and Ovarian (PLCO) Cancer Screening Trial	55-74	71,673 M + 73,816 W	400 M + 296 W	12 (median year)	1	Age, sex, race, family history of pancreatic cancer, smoking status, and randomization arm
Lukanova (2006)	Sweden	1985-2003.12	The Northern Sweden Health and Disease Cohort	29-61	33,424 M + 35,362 W	204 M + 169 W	8.2 (mean year)	1, 2, 4	Age, calendar year and smoking
Lee (2022)	Korea	2004-2017.12	The Health Examinees study	40-69	42,363 M + 80,361 W	531 M + 396 W	8.6 (mean year)	2	Education, smoking status, drinking status, family history of gastric cancer, exercise and total energy intake
Hanyuda (2017)	USA	1980-2010	The Nurses’ Health Study and the Health Professionals Follow-up Study	30-75	45,001 M + 75,812 W	1,528 M + W	32 or 26 years	4	Family history of colorectal cancer, history of colonoscopy/sigmoidoscopy, smoking in pack-years, physical activity, red and processed meat intake, alcohol consumption, current multivitamin use, regular use of aspirin, regular use of non-steroidal anti-inflammatory drugs, total energy intake, folate intake, calcium intake, and Alternate Healthy Eating Index. Additionally adjusted for menopause/postmenopausal hormone use status for women
Johansen (2009)	Sweden	1974-2004.12	The Malmö Preventive Project	50 for M and 44 for W (mean age)	22,444 M + 10,902 W	128 M + 55 W	22.1 (mean year)	1	Age, sex, smoking status, Mm-MAST category and BMI
Stolzenberg-Solomon (2008)	USA	1995-2000	The National Institutes of Health (NIH)-AARP Diet and Health Study	50-71	293,562 M + 201,473 W	429 M + 225 W	4-5 years	1	Age, smoking, race, energy, energy adjusted total fat, self-reported diabetes and sex
Jun (2022)	Korea	2003-2018.12	The National Health Insurance Service	18-99	7,695,540 M + 6,570,282 W	38,306 M + 9,002 W	13.7 (mean year)	3	Age at baseline, sex, household income, smoking status, alcohol use, and physical activity
Stolzenberg-Solomon (2013)	USA	1995-2006.12	The National Institutes of Health (NIH)-AARP Diet and Health Study	50-71	296,448 M + 205,250 W	1,206 M + W	10.5 (median year)	1	Age, smoking, energy, energy-adjusted total fat and sex
Yang (2017)	USA	2011	The National Institutes of Health (NIH)-AARP Diet and Health Study	50-71	176,789 M + 126,831 W	356 M + W	11.9 (mean year)	3	Age at baseline, sex, physical activity, cigarette smoking, alcohol consumption, history of diabetes, and red meat consumption
Pang (2019)	China	2004-2016.1	The prospective China Kadoorie Biobank (CKB) study	30-79	503,991 M + W	2,568 M + W	10 years	3	Age at baseline, education, smoking, alcohol, and total physical activity
Engeland (2005)	Norway	1963-2002.12	General Norwegian population	20-74	963,709 M + 1,038,010 W	23,615 M + 25,217 M	23 (mean year)	4	Age at measurement and birth cohort
Andreotti (2010)	USA	1993.12-2005.12	The Agricultural Health Study	NA	39,628 M + 28,319 W	125 M + 35 W	> 10 (median year)	1, 2, 5	Smoking status, diabetes and race
Otani (2005)	Japan	1990-2001.12	The Japan Public Health Center-based Prospective Study Cohort I and Cohort II	40-69	49,158 M + 53,791 W	626 M + 360 W	9.4 (mean year)	4	Age, Public Health Center areas, smoking, alcohol consumption, miso soup intake, refraining from salty foods and animal fats
Kitahara (2013)	USA	1993-2009.12	The Prostate, Lung, Colorectal, and Ovarian Cancer Screening Trial	55-74	36,912 M + 37,562 W	549 M + 417 W	11.9 (median year)	4	Age at baseline questionnaire completion, sex, study center, screening at baseline, T3, or T5 and prior to colorectal cancer diagnosis, screening adequacy and results at baseline, T3, or T5 screen and prior to colorectal cancer diagnosis, race/ethnicity, smoking status, and menopausal hormone therapy use
Hwang (2021)	Korea	2009.1-2017.12	The NHIS database maintained by the Korean NHIS	≥ 20	5,303,432 M + 4,368,509 W	21,356 M + 5,623 W	7.3 (mean year)	3	Age, sex, alcohol intake, smoking, physical activity, income status, diabetes, hypertension, dyslipidemia, liver cirrhosis and viral hepatitis
Wang (2008)	USA	1997-2005.6	The Cancer Prevention Study-II Nutrition Cohort	≥ 45	44,068 M + 51,083 W	546 M + 407 W	7.7 (median year)	4	Height, waist circumference, education, physical activity, smoking, alcohol intake, aspirin and other nonsteroidal anti-inflammatory drugs use, multivitamin use, and history of colorectal endoscopy and hormone replacement therapy use
Jiao (2010)	USA, Finland, China	1983-2006	Pooled analysis of seven prospective cohorts	58.3 (mean age)	458,070 M + 485,689 W	1,548 M + 906 W	6.9 (mean year)	1	Age, sex, cohort, smoking habit
Campbell (2016)	USA	1980-2011	The Liver Cancer Pooling Project	58.2 (mean age)	640,367 M + 929,656 W	1,481 M + 681 W	NA	3	Age, sex, study, alcohol, cigarette smoking, race, and diabetes
Kantor (2016)	Sweden	1969-2010.1	Men who underwent a compulsory conscription assessment for the Swedish military between 1969 and 1976	18.5 (mean age)	239,658 M	885 M	35 (mean year)	4	Age at conscription, erythrocyte sedimentation rate, erythrocyte volume fraction, BMI, household crowding, health status, systolic blood pressure, diastolic blood pressure, muscular strength, physical working capacity, cognitive function
Rapp (2005)	Austria	1985-2001	The Vorarlberg Health Monitoring and Promotion Program	18-94	67,447 M + 78,484 W	267 M + 183 W	10 (mean year)	1, 2, 3	Smoking status and occupational group
Han (2014)	USA	1987-2006	The Atherosclerosis Risk in Communities (ARIC) study	45-64	6,332 M + 7,569 W	151 M + 147 W	9 years	4	Race-center, age, education, height and smoking status at age 25, cigarette smoking status, alcohol consumption, physical activity at baseline, and weight change percentage from age 25 to baseline
Larsson (2005)	Sweden	1997-2004.12	The Swedish Mammography Cohort and the Cohort of Swedish Men	60 for M and 62 for W (mean age)	45,906 M + 37,147 W	75 M + 61 W	NA	1	Education, physical activity, cigarette smoking, alcohol consumption, and diabetes
Kuchiba (2012)	USA	1986.6-2004.6	The Nurses’ Health Study	NA	109,051 W	536 W	NA	4	Age, physical activity, energy-adjusted dietary and supplmental folate, vitamin D, and calcium, total calorie, red meat intake, current smoking status, pack-years of smoking before 30 years of age, alcohol intake, multivitamin use, regular aspirin use, previous sigmoidoscopy, family history of colorectal cancer in any first-degree relative, and menopausal status and postmenopausal hormone replacement therapy use
Morikawa (2013)	USA	1986-2004	The Health Professionals Follow-up Study and the Nurses’ Health Study	NA	47,684 M + 109,046 W	368 M + 493 W	NA	4	Cumulative mean physical activity, alcohol, folate, vitamin D, calcium, caloric and red meat intake, current smoking status, smoking before 30 years of age, current multivitamin use, current aspirin use, previous sigmoidoscopy, family history of colorectal cancer, and postmenopausal hormone therapy for women
Matsuo (2012)	Japan	1958-2006	Eight large-scale population-based cohort studies in Japan	≥ 35	157,927 M + 183,457 W	3,055 M + 1,924 W	11 (mean year)	4	Age, area, smoking, drinking and total energy, red meat in quartile, dietary fiber in quartile, calcium intake in quartile, folate intake in quartile and recreational physical exercise
Jung (2019)	Korea	2002.1-2010.12	The National Health Insurance Service	45.5 (mean age)	10,962,385 W	59,094 W	NA	1, 2, 3, 4	Age, smoking status, alcohol consumption frequency, physical activity times, fasting serum glucose level, fasting serum cholesterol level, comorbidity, and average insurance premium per month

W, women; M, men; 1, pancreatic cancer; 2, gastric cancer; 3, liver cancer; 4, colorectal cancer; 5, esophageal cancer.

### 3.3 Pancreatic cancer

**Fig 2:**
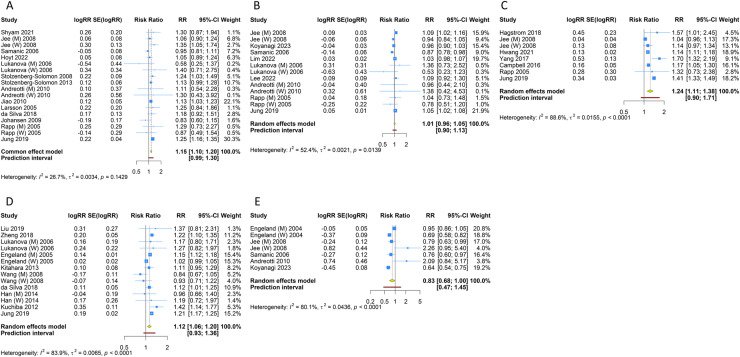
Forest plot of the association between overweight and digestive system cancers in the total population. (A) Pancreatic cancer; (B) Gastric cancer; (C) Liver cancer; (D) Colorectal cancer; (E) Esophageal cancer.

**Fig 3:**
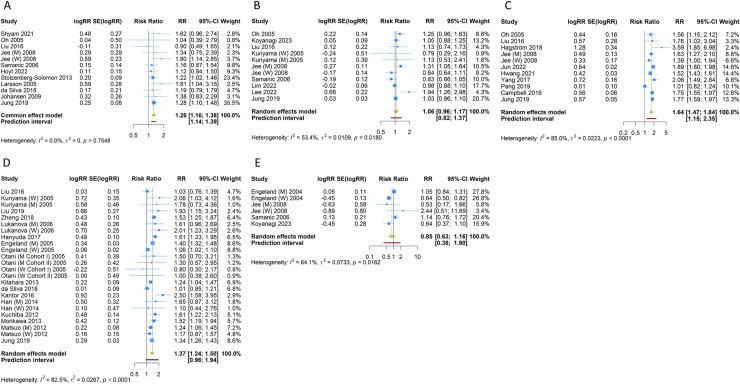
Forest plot of the association between obesity and digestive system cancers in the total population. (A) Pancreatic cancer; (B) Gastric cancer; (C) Liver cancer; (D) Colorectal cancer; (E) Esophageal cancer.

16 prospective cohort studies examined the association between overweight/obesity and pancreatic cancer. The fixed-effects pooled estimate suggested that overweight (RR [95% CI] =  1.149 [1.102-1.198]; 95% PI: 0.985-1.299; I^2^ =  26.7%, Tau^2^ =  0.0034) or obesity (RR [95% CI] =  1.262 [1.155-1.378]; 95% PI: 1.141-1.395; I^2^ =  0, Tau^2^ =  0) increased the risk of pancreatic cancer compared with normal weight (Table 2, Figs 2A and 3A). However, overweight (RR [95% CI] =  1.067 [0.991-1.149]; 95% PI: 0.976-1.167; I^2^ =  0, Tau^2^ =  0) or obesity (RR [95% CI] =  1.256 [0.995-1.584]; 95% PI: 0.861-1.831; I^2^ =  0, Tau^2^ =  0) was not associated with an increased risk of pancreatic cancer in men; while in women, overweight (RR [95% CI] =  1.237 [1.165-1.314]; 95% PI: 1.154-1.327; I^2^ =  0, Tau^2^ =  0) or obesity (RR [95% CI] =  1.306 [1.152-1.480]; 95% PI: 1.108-1.539; I^2^ =  0, Tau^2^ =  0) increased risk of pancreatic cancer (Table 2, S1–S4 Figs of S3 File).

**Table 2:** Summary risk estimates for the association between overweight/obesity and risk of digestive system cancers.

Groups	Number of studies	Meta-analysis				Heterogeneity	
		RR	95% CI	P value	95% PI	I^2^, Tau^2^	P value
Pancreatic cancer							
Obesity vs. Normal weight	11	1.262	1.155-1.378	<0.001	1.141-1.395	0%, 0	0.755
Men	4	1.256	0.995-1.584	0.055	0.861-1.831	0%, 0	0.491
Women	6	1.306	1.152-1.480	<0.001	1.108-1.539	0%, 0	0.506
Overweight vs. Normal weight	14	1.149	1.102-1.198	<0.001	0.985-1.299	26.7%, 0.0034	0.143
Men	8	1.067	0.991-1.149	0.084	0.976-1.167	0%, 0	0.528
Women	10	1.237	1.165-1.314	<0.001	1.154-1.327	0%, 0	0.912
Gastric cancer							
Obesity vs. Normal weight	9	1.059	0.960-1.169	0.249	0.815-1.377	53.4%, 0.0109	0.018
Men	5	1.178	0.915-1.517	0.203	0.581-2.388	65.6%, 0.0482	0.020
Women	5	1.054	0.845-1.314	0.644	0.601-1.846	52.4%, 0.0281	0.078
Overweight vs. Normal weight	9	1.008	0.965-1.053	0.735	0.901-1.126	52.4%, 0.0021	0.014
Men	6	1.024	0.899-1.167	0.722	0.740-1.417	59.5%, 0.0115	0.030
Women	6	1.041	1.013-1.070	0.004	0.806-1.230	38.3%, 0.0043	0.151
Liver cancer							
Obesity vs. Normal weight	10	1.642	1.466-1.839	<0.001	1.143-2.358	85.0%, 0.0223	<0.001
Men	6	1.719	1.475-2.003	<0.001	1.099-2.690	86.4%, 0.0243	<0.001
Women	6	1.821	1.724-1.924	<0.001	1.486-2.146	38.8%, 0.0034	0.147
Overweight vs. Normal weight	7	1.237	1.112-1.377	<0.001	0.888-1.725	88.6%, 0.0155	<0.001
Men	5	1.093	1.058-1.129	<0.001	0.931-1.332	47.9%, 0.0027	0.104
Women	4	1.256	1.127-1.400	<0.001	0.891-1.772	79.2%, 0.0086	0.002
Colorectal cancer							
Obesity vs. Normal weight	16	1.366	1.242-1.503	<0.001	0.959-1.945	82.5%, 0.0267	<0.001
Men	11	1.405	1.337-1.476	<0.001	1.329-1.485	0%, 0	0.448
Women	15	1.305	1.160-1.468	<0.001	0.890-1.912	80.8%, 0.0285	<0.001
Overweight vs. Normal weight	10	1.124	1.056-1.197	<0.001	0.931-1.357	83.9%, 0.0065	<0.001
Men	6	1.148	1.119-1.177	<0.001	0.905-1.381	45.1%, 0.0047	0.105
Women	10	1.146	1.044-1.258	0.004	0.878-1.496	86.4%, 0.0116	<0.001
Esophageal cancer							
Obesity vs. Normal weight	4	0.855	0.631-1.157	0.309	0.384-1.904	64.1%, 0.0733	0.016
Men	3	1.048	0.865-1.271	0.631	0.687-1.600	0%, 0	0.464
Women	2	0.990	0.290-3.387	0.988	–	63.4%, 0.5682	0.098
Overweight vs. Normal weight	5	0.826	0.681-1.002	0.052	0.457-1.495	80.1%, 0.0436	<0.001
Men	4	0.877	0.726-1.060	0.174	0.510-1.507	59.8%, 0.0196	0.058
Women	2	1.153	0.364-3.649	0.809	–	85.4%, 0.6012	0.009

### 3.4 Gastric cancer

12 prospective cohort studies assessed gastric cancer in overweight/obesity and normal weight groups. The pooled RR was 1.008 (95% CI =  0.965-1.053; 95% PI: 0.901-1.126; I^2^ =  52.4%, Tau^2^ =  0.0021) and 1.059 (95% CI =  0.960-1.169; 95% PI: 0.815-1.377; I^2^ =  53.4%, Tau^2^ =  0.0109) for overweight and obesity, respectively, indicating overweight and obesity did not increase the risk of gastric cancer compared with normal weight (Table 2, Figs 2B and 3B). In men, overweight (RR [95% CI] =  1.024 [0.899-1.167]; 95% PI: 0.740-1.417; I^2^ =  59.5%, Tau^2^ =  0.0115) or obesity (RR [95% CI] =  1.178 [0.915-1.517]; 95% PI: 0.581-2.388; I^2^ =  65.6%, Tau^2^ =  0.0482) was associated with no higher risk of gastric cancer. Overweight women had an increased risk of gastric cancer compared with normal-weight women (RR [95% CI] =  1.041 [1.013-1.070]; 95% PI: 0.806-1.230; I^2^ =  38.3%, Tau^2^ =  0.0043), while obese women had no increased risk of gastric cancer (RR [95% CI] =  1.054 [0.845-1.314]; 95% PI: 0.601-1.846; I^2^ =  52.4%, Tau^2^ =  0.0281) (Table 2, S1–S4 Figs of S3 File).

### 3.5 Liver cancer

11 prospective cohort studies examined the link between being overweight/obese and the incidence of liver cancer. The results showed that compared with normal weight, overweight (RR [95% CI] =  1.237 [1.112-1.377]; 95% PI: 0.888-1.725; I^2^ =  88.6%, Tau^2^ =  0.0155) or obesity (RR [95% CI] =  1.642 [1.466-1.839]; 95% PI: 1.143-2.358; I^2^ =  85.0%, Tau^2^ =  0.0223) increased the risk of liver cancer in the total population (Table 2, Figs 2C and 3C). Overweight and obesity were associated with higher risks of liver cancer in both men and women (Men: overweight: RR [95% CI] =  1.093 [1.058-1.129]; 95% PI: 0.931-1.332; I^2^ =  47.9%, Tau^2^ =  0.0027; obesity: RR [95% CI] =  1.719 [1.475-2.003]; 95% PI: 1.099-2.690; I^2^ =  86.4%, Tau^2^ =  0.0243. Women: overweight: RR [95% CI] =  1.256 [1.127-1.400]; 95% PI: 0.891-1.772; I^2^ =  79.2%, Tau^2^ =  0.0086; obesity: RR [95% CI] =  1.821 [1.724-1.924]; 95% PI: 1.486-2.146; I^2^ =  38.8%, Tau^2^ =  0.0034) (Table 2, S1–S4 Figs of S3 File).

### 3.6 Colorectal cancer

17 prospective cohort studies have investigated the relationship between overweight/obesity and the risk of colorectal cancer. The result indicated that compared with normal weight, overweight (RR [95% CI] =  1.124 [1.056-1.197]; 95% PI: 0.931-1.357; I^2^ =  83.9%, Tau^2^ =  0.0065) or obesity (RR [95% CI] =  1.366 [1.242-1.503]; 95% PI: 0.959-1.945; I^2^ =  82.5%, Tau^2^ =  0.0267) increased the risk of colorectal cancer in the total population (Table 2, Figs 2D and 3D). Overweight and obesity were related to higher risks of colorectal cancer in both men and women (Men: overweight: RR [95% CI] =  1.148 [1.119-1.177]; 95% PI: 0.905-1.381; I^2^ =  45.1%, Tau^2^ =  0.0047; obesity: RR [95% CI] =  1.405 [1.337-1.476]; 95% PI: 1.329-1.485; I^2^ =  0, Tau^2^ =  0. Women: overweight: RR [95% CI] =  1.146 [1.044-1.258]; 95% PI: 0.878-1.496, I^2^ =  86.4%, Tau^2^ =  0.0116; obesity: RR [95% CI] =  1.305 [1.160-1.468]; 95% PI: 0.890-1.912; I^2^ =  80.8%, Tau^2^ =  0.0285) (Table 2, S1–S4 Figs of S3 File).

### 3.7 Esophageal cancer

Esophageal cancer was evaluated in 5 prospective cohort studies. Compared with normal weight, overweight (RR [95% CI] =  0.826 [0.681-1.002]; 95% PI: 0.457-1.495; I^2^ =  80.1%, Tau^2^ =  0.0436) or obesity (RR [95% CI] =  0.855 [0.631-1.157]; 95% PI: 0.384-1.904; I^2^ =  64.1%, Tau^2^ =  0.0733) did not increase the risk of esophageal cancer in the total population (Table 2, Figs 2E and 3E). No significant association of overweight/obesity and esophageal cancer was observed in both men and women (Men: overweight: RR [95% CI] =  0.877 [0.726-1.060]; 95% PI: 0.510-1.507; I^2^ =  59.8%, Tau^2^ =  0.0196; obesity: RR [95% CI] =  1.048 [0.865-1.271]; 95% PI: 0.687-1.600; I^2^ =  0, Tau^2^ =  0. Women: overweight: RR [95% CI] =  1.153 [0.364-3.649]; I^2^ =  85.4%, Tau^2^ =  0.6012; obesity: RR [95% CI] =  0.990 [0.290-3.387]; I^2^ =  63.4%, Tau^2^ =  0.5682) (Table 2, S1–S4 Figs of S3 File).

### 3.8 Trial sequential analysis results

**Fig 4:**
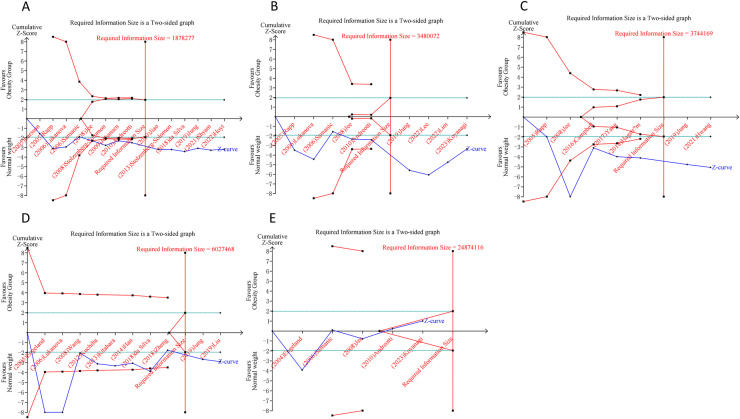
Trial sequential analysis of the association between overweight and digestive system cancers in the total population. (A) Pancreatic cancer; (B) Gastric cancer; (C) Liver cancer; (D) Colorectal cancer; (E) Esophageal cancer. Uppermost and lowermost red curves represent trial sequential monitoring boundary lines for benefit and harm, respectively. Inner red lines represent the futility boundary. Blue line represents evolution of cumulative Z-score. Horizontal green lines represent the conventional boundaries for statistical significance. Cumulative Z-curve crossing the trial sequential monitoring boundary or the RIS boundary provides firm evidence of effect.

**Fig 5:**
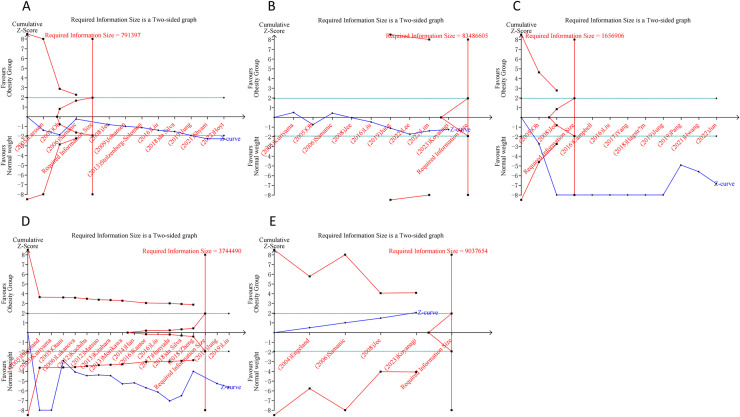
Trial sequential analysis of the association between obesity and digestive system cancers in the total population. (A) Pancreatic cancer; (B) Gastric cancer; (C) Liver cancer; (D) Colorectal cancer; (E) Esophageal cancer. Uppermost and lowermost red curves represent trial sequential monitoring boundary lines for benefit and harm, respectively. Inner red lines represent the futility boundary. Blue line represents evolution of cumulative Z-score. Horizontal green lines represent the conventional boundaries for statistical significance. Cumulative Z-curve crossing the trial sequential monitoring boundary or the RIS boundary provides firm evidence of effect.

In the case of overweight and DSCs, the cumulative Z-curves for pancreatic, liver, and colorectal cancers distinctly surpassed both the trial sequential monitoring boundaries and the RIS boundaries. Meanwhile, the Z-curve for gastric cancer only exceeded the RIS threshold, and the Z-curve for esophageal cancer failed to cross either the trial sequential monitoring boundary or the RIS threshold. This suggests that conclusive evidence is available regarding the impact of overweight on pancreatic, liver, colorectal, and gastric cancers, while conclusive results for esophageal cancer remain elusive, potentially due to false positives in the analysis (Fig 4). For the association of obesity and DSC, the cumulative Z-curves for pancreatic, liver, and colorectal cancers have markedly exceeded the RIS threshold. However, the Z-curves for gastric and esophageal cancers have failed to surpass both the trial sequential monitoring boundaries and the RIS threshold. Thses findings suggested that conclusive evidence has been established regarding the impact of obesity on pancreatic, liver, and colorectal cancers, while the outcomes for gastric and esophageal cancers remain uncertain, likely due to the influence of false positives in the obesity and gastric and esophageal cancer analyses (Fig 5).

### 3.9 Publication bias and sensitivity analysis

The sensitivity analyses alongside tests for publication bias were performed for the outcomes of pancreatic, cancer, cancer and colorectal cancers which included ≥  8 studies. Through the application of the trim-and-fill method, an assessment and correction for publication bias were executed. Funnel plot region contained missing studies were observed in the association between overweight and liver or colorectal cancer, or between obesity and pancreatic, gastric or colorectal cancer. After correcting for publication bias, the outcomes remained consistent with prior findings, affirming the dependability of these results. The funnel plots were presented in S5 and S6 Figs of S3 File. To ascertain the robustness of our findings, a sensitivity analysis employing a leave-one-out approach was conducted. The results pinpointed the study by Engeland et al. (2005) as a potential source of the observed high heterogeneity in the relationship between being overweight/obese and colorectal cancer. Similarly, the studies conducted by Jung et al. and Pang et al. were identified as likely contributors to the notable heterogeneity observed in the association between overweight/obesity and liver cancer, respectively (S7 and S8 Figs of S3 File).

## 4. Discussion

Over the past four decades, the global incidence of overweight and obesity has consistently increased [73]. The efforts to prevent the obesity epidemic has failed despite the adjustable factor of body weight. Substantial evidence supports a direct correlation between overweight/obesity and cancer of the pancreas, kidney, gastric cardia, breast (postmenopausal), endometrium, ovary, colon-rectum, gallbladder, liver, thyroid, oesophagus (adenocarcinoma), meningioma and multiple myeloma [74,75]. In this investigation, we performed a meta-analysis of large, high-quality prospective cohort studies to evaluate the link between overweight/obesity and the risk of developing DSC. The final results showed that overweight/obesity heightened the likelihood of liver and colorectal cancers relative to normal weight, while being overweight or obese was linked to greater risks of pancreatic and gastric cancers in women, respectively. No significant association was found between overweight/obesity and the risk of esophageal cancer.

Pancreatic cancer remains an understudied area in oncology, and the understanding of its etiologic factors is still in its infancy compared with other cancers [76]. Obesity stands out as one of the limited identifiable changeable risk elements linked to pancreatic cancer [77]. Overweight and obesity cause insulin resistance and eventually diabetes [78]. Prospective epidemiological studies have showed that associations between biomarkers indicative of insulin resistance and an increased risk of pancreatic cancer have been identified [79,80]. Previous researches have shown that insulin can prompt the proliferation of pancreatic cancer cells, and that insulin resistance accelerates the progression of pancreatic ductal carcinoma in animal studies [81,82]. Researches have also demonstrated that the therapeutic application of certain antidiabetic agents, notably metformin, has been correlated with a diminished incidence and retardation of pancreatic cancer progression. Conversely, the use of insulin and insulin secretagogues is associated with a heightened likelihood of this cancer’s emergence [83,84]. Our study demonstrated a gender-specific association, where overweight/obesity was observed to escalate the risk of pancreatic cancer exclusively in females. The underlying processes connecting overweight/obesity to the risk of pancreatic cancer have yet to be completely understood. However, study has linked hormones, including sex hormones, to a heightened incidence of pancreatic cancer [85]. This implies that the relationship between excess weight or obesity and the probability of contracting pancreatic cancer could differ according to sex.

The biological processes underpinning the promotion of gastric cancer by overweight or obesity remain to be elucidated. The abnormal fat deposition might lead to increased insulin-like growth factors, increase of estrogen, molecular changes by hyperinsulinemia, and adipocytokine imbalance based on previous studies [86,87]. Furthermore, the abnormal build-up of fat cells triggers pro-inflammatory responses via inflammatory cytokines, such as interleukin-6 and tumor necrosis factors. This activity leads to a persistent, low-grade inflammation, compounded by oxidative stress, which is widely acknowledged as a promoter of cancer development [88]. However, the evidence connecting excess weight/obesity with the risk of gastric cancer has yielded inconsistent outcomes. Research has identified that obesity is associated with a 36% increase in the risk for the development of gastric cancer, while being overweight does so by 21% [89]. Subsequent meta-analyses, encompassing a broader array of prospective studies, have not succeeded in establishing a statistically significant link between obesity and gastric cancer [90,91]. In our examination of gastric cancer risk, we observed an elevated risk exclusively in overweight females, while no substantial correlation was found between obesity and an increased incidence of gastric cancer. The TSA results showed that the cumulative Z-curve for obesity and gastric cancer did not cross the RIS boundary, indicating that the limited sample size contributed to the absence of statistically significant results. Additionally, the male subgroup analysis predominantly reviewed studies published from 2005 to 2008, suggesting a need for updated combined findings. Therefore, future research should include larger and more recent prospective cohort studies to further validate and refine our findings.

The present research has identified a positive correlation between being overweight or obese and the increased likelihood of developing liver and colorectal cancer. The association between enhanced risk of liver cancer and excess body weight is predominantly attributed to the pathophysiological mechanisms underlying non-alcoholic fatty liver disease (NAFLD) [92]. NAFLD constitutes a range of hepatic disorders characterized by the accumulation of excess fat within liver cells, extending from simple fatty liver to nonalcoholic steatohepatitis (NASH), which may advance to cirrhosis [93]. The presence of NAFLD often leads to mild, persistent liver inflammation and insulin resistance, resulting in lipid peroxidation and the generation of oxidative stress through free radicals [93]. Such processes contribute to cellular dysfunction and oxidative damage to the DNA, ultimately triggering the development of liver cancer [94]. The precise mechanisms connecting excess weight or obesity to liver cancer are not entirely clear, but it’s believed that changes in gut microbial metabolites, insulin resistance, and the generation of cytokines that may promote tumors, along with liver inflammation, play significant roles [95]. The precise biological pathways connecting excessive body weight or obesity to a heightened risk of colorectal cancer remain to be fully elucidated [96]. Evidence points to insulin resistance, immune response dysregulation, and systemic inflammation as critical intermediaries, with microbial imbalances also potentially contributing [97]. Moreover, obesity leads to alterations in mucosal metabolism through glycolytic and adipogenic routes and affects the functioning of adenosine monophosphate-activated protein kinase (AMPK) and sirtuins [98].Recent findings indicated that adipocytes within the tumor microenvironment undergo phenotypic transformations, which enhance the proliferation and dissemination of colorectal cancer through the release of inflammatory cytokines, alongside factors that stimulate growth and angiogenesis [99].

Our analysis yielded no significant correlation between excess weight/obesity and the incidence of esophageal cancer. ESCC and EADC, as two prevalent but histologically distinct forms of esophageal cancer, arise from diverse risk factors. Factors contributing to the risk of ESCC include tobacco use, alcohol intake, a diet lacking in fruits and vegetables, diet structure, betel intake, pickled food, foods and drinks consumed at high temperatures, micronutrients, socioeconomic status, among others [100], while smoking tobacco, Barrett’s esophagus, and gastroesophageal reflux disease have emerged as potential risk factors implicated in the etiology of EADC [101]. Additionally, the utility of BMI as an accurate measure of individual body composition is questionable, potentially contributing to the inconclusive findings. Given that most studies included in our analysis on the association between overweight/obesity and esophageal cancer were published between 2004 and 2010, and the TSA analysis suggested that the existing results require larger sample sizes for validation, comprehensive analyses with larger and more recent studies are needed to support our findings on esophageal cancer. Furthermore, beyond BMI, the relationship between other anthropometric measures, such as waist-to-hip ratio, and esophageal cancer also warrants further investigation.

Our study is subject to several notable limitations that warrant acknowledgment. First, variations in the methodology for obtaining height and weight data were observed across the studies reviewed. Specifically, discrepancies arose between studies that relied on self-reported height and weight for BMI calculations and those where measurements were directly obtained by trained personnel. This may lead to high heterogeneity between studies. Second, the consistency of outcomes across different investigations may be constrained by variations in the timing of BMI measurement, disparate inclusion and exclusion criteria, and divergent adjustments for covariates. Third, inconsistencies in reference groups across publications were noted, with these groups defined by varying BMI ranges, including but not limited to 18.5-22.9 kg/m^2^, 18.5-24.9 kg/m^2^, 18.5-23.0 kg/m^2^, and 23.0-24.9 kg/m^2^. Fourth, the TSA results suggested that the associations between overweight/obesity and esophageal cancer, as well as obesity and gastric cancer, may be uncertain and subject to false-positive errors. This could be attributed to the relatively small sample sizes included in the analysis. Although positive results for these associations were not observed in our study, further research with larger sample sizes is necessary to validate and refine these findings. Fifth, our meta-analysis was exclusively focused on employing BMI as the metric for defining overweight and obesity. Future studies could incorporate other anthropometric measurements, such as waist-to-hip ratio, to enhance the scope beyond BMI.

## 5. Conclusion

In conclusion, our meta-analysis demonstrated that compared with normal weight, overweight/obesity increased the risks of liver cancer and colorectal cancer in men and women. In women, the relationship between overweight/obesity and an augmented risk for pancreatic cancer was demonstrated, while a significant correlation was also observed between overweight and an increased risk of gastric cancer. These findings underscore the need for effective strategies to prevent and manage overweight and obesity, thereby reducing the incidence of these cancers. Healthcare providers should consider weight management as a key component of cancer prevention strategies and patient education.

## Supporting information

S1 Checklist PRISMA 2020 checklist. (PDF)

S1 File Search strategy. (DOCX)

S2 File Risk of bias and quality assessments for each study. (DOCX)

S3 File Subgroup analysis, publication bias and sensitivity analysis. (DOCX)

S1 Data Studies excluded from the analyses with the reasons for exclusion. (XLSX)

S2 Data Data extracted from the included studies for meta-analysis. (XLSX)
