# Supplementary material for: Association of overweight/obesity and digestive system cancers: A meta-analysis and trial sequential analysis of prospective cohort studies
Source: PLoS One. 2025 Apr 1;20(4):e0318256. doi: 10.1371/journal.pone.0318256 (PMC11960891; doi:10.1371/journal.pone.0318256)
Supplement: S1 File — (DOCX) [file pone.0318256.s002.docx]

**PubMed 8633**

#1 overweight OR obesity OR obese OR adiposity OR (body mass index) OR BMI

#2 ((digestive system cancer) OR (liver cancer) OR (liver neoplasm) OR (hepatic neoplasm) OR (hepatic cancer) OR (hepatocellular cancer) OR (gastric cancer) OR (stomach neoplasm) OR (gastric neoplasm) OR (gastric neoplasms) OR (stomach cancer) OR (esophageal neoplasm) OR (esophagus neoplasm) OR (esophagus neoplasms) OR (esophagus cancer) OR (esophageal cancer) OR (pancreatic neoplasm) OR (pancreas neoplasms) OR (pancreas neoplasm) OR (pancreas cancer) OR (pancreatic cancer) OR (colorectal neoplasm) OR (colorectal tumor) OR (colorectal cancer) OR (colorectal carcinoma) OR (colorectal carcinomas))

#3 (cohort study) OR (concurrent study) OR (incidence study)

#4 #1 AND #2 AND #3

**Web of Science 5337**

#1 TS=(overweight OR obesity OR obese OR adiposity OR (body mass index) OR BMI)

#2 TS=((digestive system cancer) OR (liver cancer) OR (liver neoplasm) OR (hepatic neoplasm) OR (hepatic cancer) OR (hepatocellular cancer) OR (gastric cancer) OR (stomach neoplasm) OR (gastric neoplasm) OR (gastric neoplasms) OR (stomach cancer) OR (esophageal neoplasm) OR (esophagus neoplasm) OR (esophagus neoplasms) OR (esophagus cancer) OR (esophageal cancer) OR (pancreatic neoplasm) OR (pancreas neoplasms) OR (pancreas neoplasm) OR (pancreas cancer) OR (pancreatic cancer) OR (colorectal neoplasm) OR (colorectal tumor) OR (colorectal cancer) OR (colorectal carcinoma) OR (colorectal carcinomas))

#3 TS=((cohort study) OR (concurrent study) OR (incidence study))

#4 #1 AND #2 AND #3

**Embase 5759**

#1 'overweight'/exp OR 'obesity'/exp OR obese OR 'adiposity'/exp OR 'body mass index'/exp OR 'bmi'/exp

#2 'digestive system cancer':ti,ab,kw OR 'liver cancer':ti,ab,kw OR 'liver neoplasm':ti,ab,kw OR 'hepatic neoplasm':ti,ab,kw OR 'hepatic cancer':ti,ab,kw OR 'hepatocellular cancer':ti,ab,kw OR 'gastric cancer':ti,ab,kw OR 'stomach neoplasm':ti,ab,kw OR 'gastric neoplasm':ti,ab,kw OR 'gastric neoplasms':ti,ab,kw OR 'stomach cancer':ti,ab,kw OR 'esophageal neoplasm':ti,ab,kw OR 'esophagus neoplasm':ti,ab,kw OR 'esophagus neoplasms':ti,ab,kw OR 'esophagus cancer':ti,ab,kw OR 'esophageal cancer':ti,ab,kw OR 'pancreatic neoplasm':ti,ab,kw OR 'pancreas neoplasms':ti,ab,kw OR 'pancreas neoplasm':ti,ab,kw OR 'pancreas cancer':ti,ab,kw OR 'pancreatic cancer':ti,ab,kw OR 'colorectal neoplasm':ti,ab,kw OR 'colorectal tumor':ti,ab,kw OR 'colorectal cancer':ti,ab,kw OR 'colorectal carcinoma':ti,ab,kw OR 'colorectal carcinomas':ti,ab,kw

#3 ('cohort'/exp OR cohort) AND ('study'/exp OR study) OR (concurrent AND ('study'/exp OR study)) OR (('incidence'/exp OR incidence) AND ('study'/exp OR study))

#4 #1 AND #2 AND #3

**The Cochrane Library 579**

#1 (overweight OR obesity OR obese OR adiposity OR (body mass index) OR BMI):ti,ab,kw

#2 ((digestive system cancer) OR (liver cancer) OR (liver neoplasm) OR (hepatic neoplasm) OR (hepatic cancer) OR (hepatocellular cancer) OR (gastric cancer) OR (stomach neoplasm) OR (gastric neoplasm) OR (gastric neoplasms) OR (stomach cancer) OR (esophageal neoplasm) OR (esophagus neoplasm) OR (esophagus neoplasms) OR (esophagus cancer) OR (esophageal cancer) OR (pancreatic neoplasm) OR (pancreas neoplasms) OR (pancreas neoplasm) OR (pancreas cancer) OR (pancreatic cancer) OR (colorectal neoplasm) OR (colorectal tumor) OR (colorectal cancer) OR (colorectal carcinoma) OR (colorectal carcinomas)):ti,ab,kw (Word variations have been searched)

#3 (cohort study) OR (concurrent study) OR (incidence study)

#4 #1 AND #2 AND #3
