# Supplementary material for: Association of overweight/obesity and digestive system cancers: A meta-analysis and trial sequential analysis of prospective cohort studies
Source: PLoS One. 2025 Apr 1;20(4):e0318256. doi: 10.1371/journal.pone.0318256 (PMC11960891; doi:10.1371/journal.pone.0318256)
Supplement: S2 File — (DOCX) [file pone.0318256.s003.docx]

| **Table S1.** The Newcastle-Ottawa quality assessment scale of the included studies. | | | | | | | | | | | | |
| --- | --- | --- | --- | --- | --- | --- | --- | --- | --- | --- | --- | --- |
| Study | Selection | | | |  | Comparability | |  | Assessment of outcome | | | Total score |
|  | Representativeness of exposure arm(s) | Selection of the comparative arm(s) | Origin of exposure source | Demonstration that outcome of interest was not present at start of study |  | Studies controlling the most important factors | Studies controlling the other main factors |  | Assessment of outcome with independency | Adequacy of follow-up length | Lost to follow-up acceptable |  |
|  |  |  |  |  |  |  |  |  |  |  |  |  |
| Shyam (2021) | 1 | 1 | 1 | 1 |  | 1 | 1 |  | 1 | 1 | 1 | 9 |
| Oh (2005) | 1 | 1 | 1 | 1 |  | 1 | 1 |  | 1 | 1 | 1 | 9 |
| Koyanagi (2023) | 1 | 1 | 0 | 1 |  | 1 | 1 |  | 1 | 1 | 1 | 8 |
| Liu (2016) | 1 | 1 | 1 | 1 |  | 1 | 1 |  | 1 | 1 | 1 | 9 |
| Kuriyama (2005) | 0 | 1 | 0 | 1 |  | 1 | 1 |  | 1 | 1 | 1 | 7 |
| Liu (2019) | 1 | 1 | 0 | 1 |  | 1 | 1 |  | 1 | 1 | 1 | 8 |
| Engeland (2004) | 1 | 1 | 1 | 1 |  | 1 | 1 |  | 1 | 1 | 1 | 9 |
| Zheng (2018) | 1 | 1 | 0 | 1 |  | 1 | 1 |  | 1 | 1 | 1 | 8 |
| Hagström (2018) | 1 | 1 | 1 | 1 |  | 1 | 1 |  | 1 | 1 | 1 | 9 |
| Jee (2008) | 1 | 1 | 0 | 1 |  | 1 | 1 |  | 1 | 1 | 1 | 8 |
| Samanic (2006) | 0 | 1 | 1 | 1 |  | 1 | 1 |  | 1 | 1 | 1 | 8 |
| Lim (2022) | 1 | 1 | 1 | 1 |  | 1 | 1 |  | 1 | 1 | 1 | 9 |
| Hoyt (2022) | 1 | 1 | 1 | 1 |  | 1 | 1 |  | 1 | 1 | 1 | 9 |
| Lukanova (2006) | 1 | 1 | 0 | 1 |  | 1 | 1 |  | 1 | 1 | 1 | 8 |
| Jun (2022) | 1 | 1 | 1 | 1 |  | 1 | 1 |  | 1 | 1 | 1 | 9 |
| Hwang (2021) | 1 | 1 | 1 | 1 |  | 1 | 1 |  | 1 | 1 | 1 | 9 |
| Lee (2022) | 1 | 1 | 1 | 1 |  | 1 | 1 |  | 1 | 1 | 1 | 9 |
| Hanyuda (2017) | 1 | 1 | 0 | 1 |  | 1 | 1 |  | 1 | 1 | 1 | 8 |
| Stolzenberg-Solomon (2008) | 1 | 1 | 0 | 1 |  | 1 | 1 |  | 1 | 1 | 1 | 8 |
| Stolzenberg-Solomon (2013) | 1 | 1 | 0 | 1 |  | 1 | 1 |  | 1 | 1 | 1 | 8 |
| Yang (2017) | 1 | 1 | 0 | 1 |  | 1 | 1 |  | 1 | 1 | 1 | 8 |
| Pang (2019) | 1 | 1 | 1 | 1 |  | 1 | 1 |  | 1 | 1 | 1 | 9 |
| Engeland (2005) | 1 | 1 | 0 | 1 |  | 1 | 1 |  | 1 | 1 | 1 | 8 |
| Andreotti (2010) | 1 | 1 | 0 | 1 |  | 1 | 1 |  | 1 | 1 | 0 | 7 |
| Otani (2005) | 1 | 1 | 0 | 1 |  | 1 | 1 |  | 1 | 1 | 1 | 8 |
| Kitahara (2013) | 1 | 1 | 0 | 1 |  | 1 | 1 |  | 1 | 1 | 1 | 8 |
| Wang (2008) | 1 | 1 | 0 | 1 |  | 1 | 1 |  | 1 | 1 | 1 | 8 |
| Jiao (2010) | 1 | 1 | 0 | 1 |  | 1 | 1 |  | 1 | 1 | 1 | 8 |
| Campbell (2016) | 1 | 1 | 0 | 1 |  | 1 | 1 |  | 1 | 1 | 1 | 8 |
| Larsson (2005) | 1 | 1 | 0 | 1 |  | 1 | 1 |  | 1 | 1 | 1 | 8 |
| da Silva (2018) | 1 | 1 | 0 | 1 |  | 1 | 1 |  | 1 | 1 | 1 | 8 |
| Johansen (2009) | 1 | 1 | 1 | 1 |  | 1 | 1 |  | 1 | 1 | 1 | 9 |
| Kantor (2016) | 1 | 1 | 1 | 1 |  | 1 | 1 |  | 1 | 1 | 1 | 9 |
| Rapp (2005) | 1 | 1 | 1 | 1 |  | 1 | 1 |  | 1 | 1 | 1 | 9 |
| Han (2014) | 0 | 1 | 1 | 1 |  | 1 | 1 |  | 1 | 1 | 1 | 8 |
| Kuchiba (2012) | 1 | 1 | 0 | 1 |  | 1 | 1 |  | 1 | 1 | 0 | 7 |
| Morikawa (2013) | 1 | 1 | 1 | 1 |  | 1 | 1 |  | 1 | 1 | 1 | 9 |
| Matsuo (2012) | 1 | 1 | 0 | 1 |  | 1 | 1 |  | 1 | 1 | 1 | 8 |
| Jung (2019) | 1 | 1 | 1 | 1 |  | 1 | 1 |  | 1 | 1 | 1 | 9 |
