# Supplementary material for: Association of overweight/obesity and digestive system cancers: A meta-analysis and trial sequential analysis of prospective cohort studies
Source: PLoS One. 2025 Apr 1;20(4):e0318256. doi: 10.1371/journal.pone.0318256 (PMC11960891; doi:10.1371/journal.pone.0318256)

**Fig S1.** Subgroup analysis of the association between overweight and digestive system cancers in men. (A) Pancreatic cancer; (B) Gastric cancer; (C) Liver cancer; (D) Colorectal cancer; (E) Esophageal cancer.

**Fig S2.** Subgroup analysis of the association between overweight and digestive system cancers in women. (A) Pancreatic cancer; (B) Gastric cancer; (C) Liver cancer; (D) Colorectal cancer; (E) Esophageal cancer.


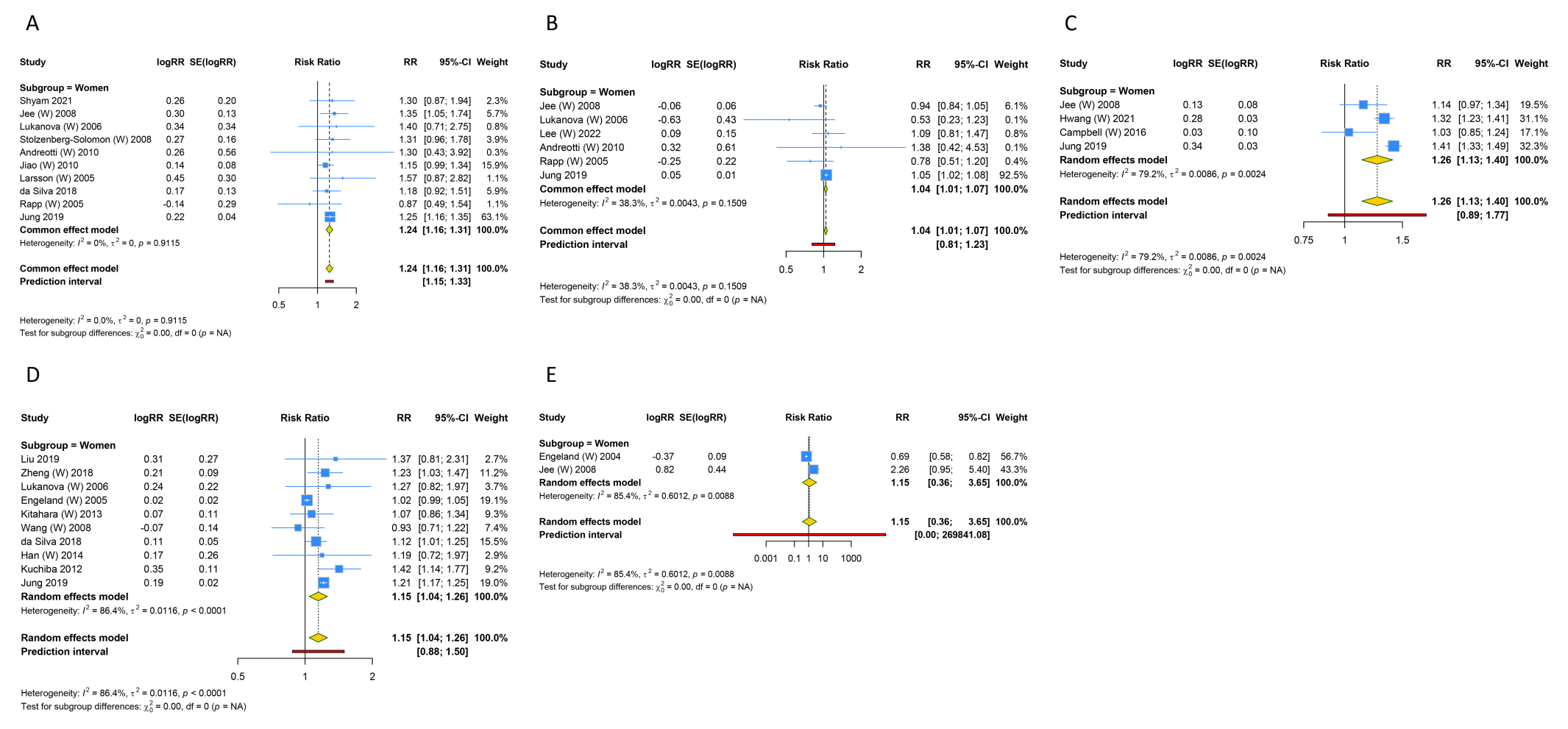


**Fig S3.** Subgroup analysis of the association between obesity and digestive system cancers in men. (A) Pancreatic cancer; (B) Gastric cancer; (C) Liver cancer; (D) Colorectal cancer; (E) Esophageal cancer.

**Fig S4.** Subgroup analysis of the association between obesity and digestive system cancers in women. (A) Pancreatic cancer; (B) Gastric cancer; (C) Liver cancer; (D) Colorectal cancer; (E) Esophageal cancer.


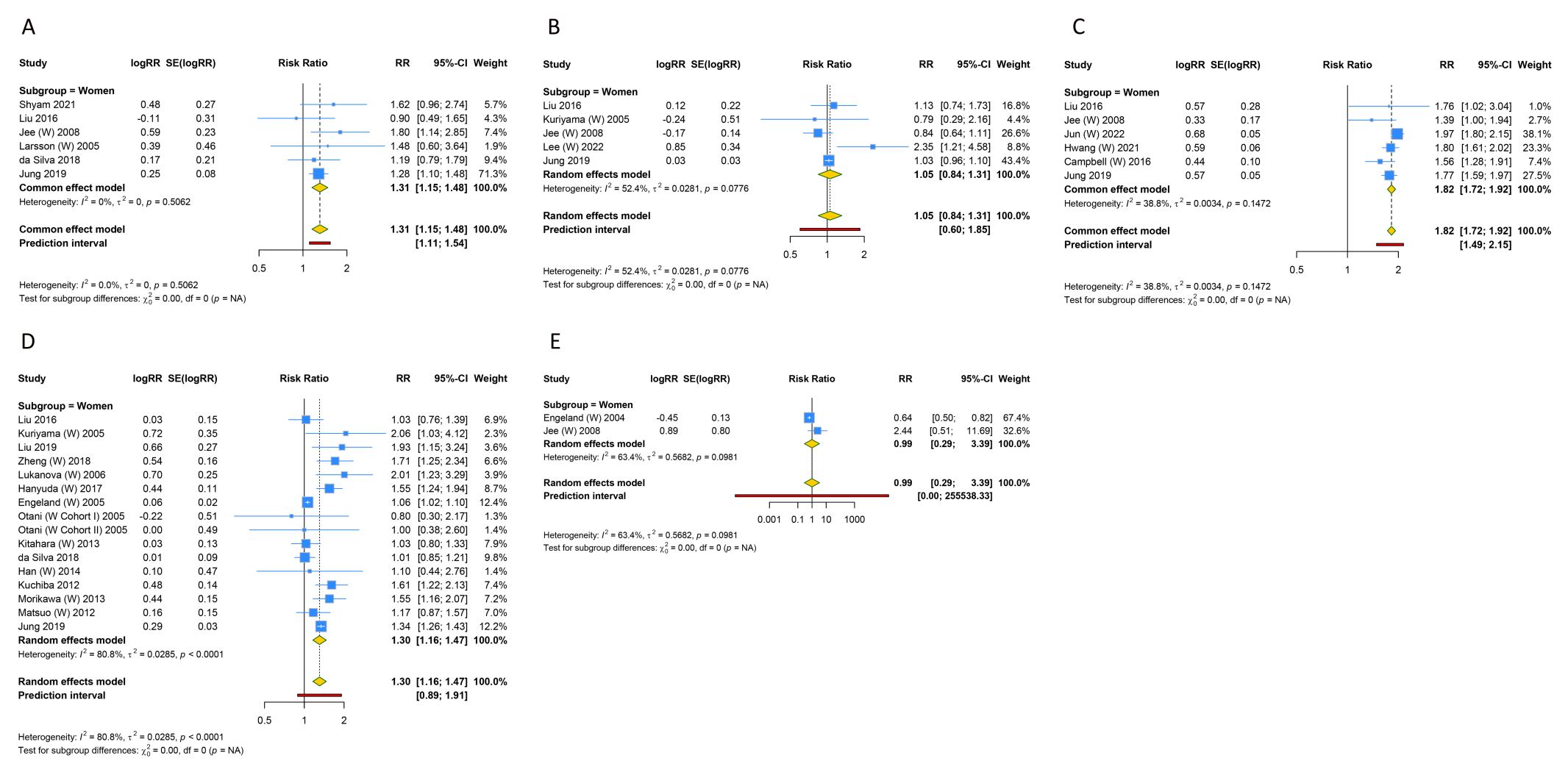


**Fig S5.** Funnel plot of trim-and-fill method on the association between overweight and digestive system cancers. (A) Pancreatic cancer; (B) Gastric cancer; (C) Liver cancer; (D) Colorectal cancer.


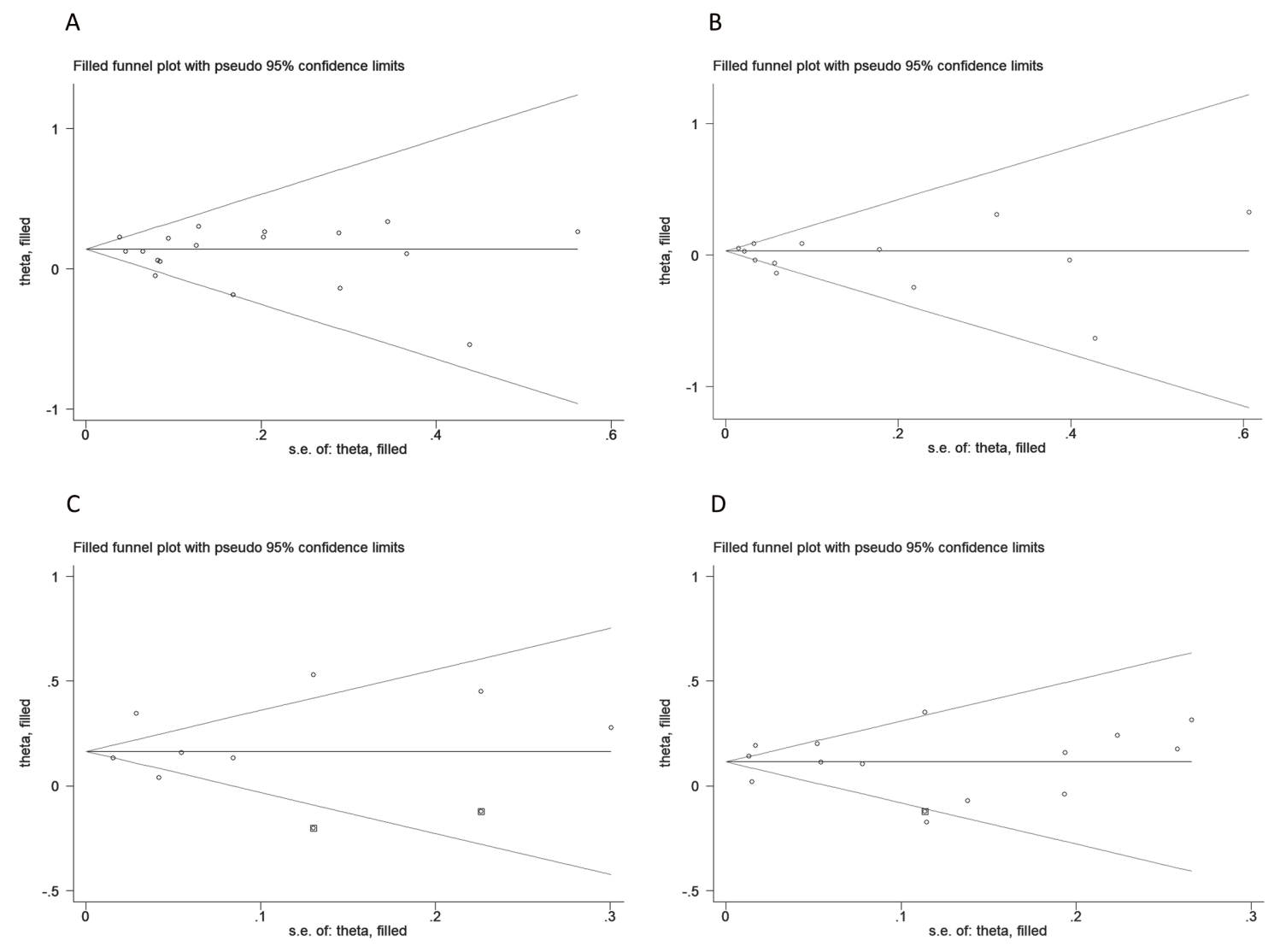


**Fig S6.** Funnel plot of trim-and-fill method on the association between obesity and digestive system cancers. (A) Pancreatic cancer; (B) Gastric cancer; (C) Liver cancer; (D) Colorectal cancer.


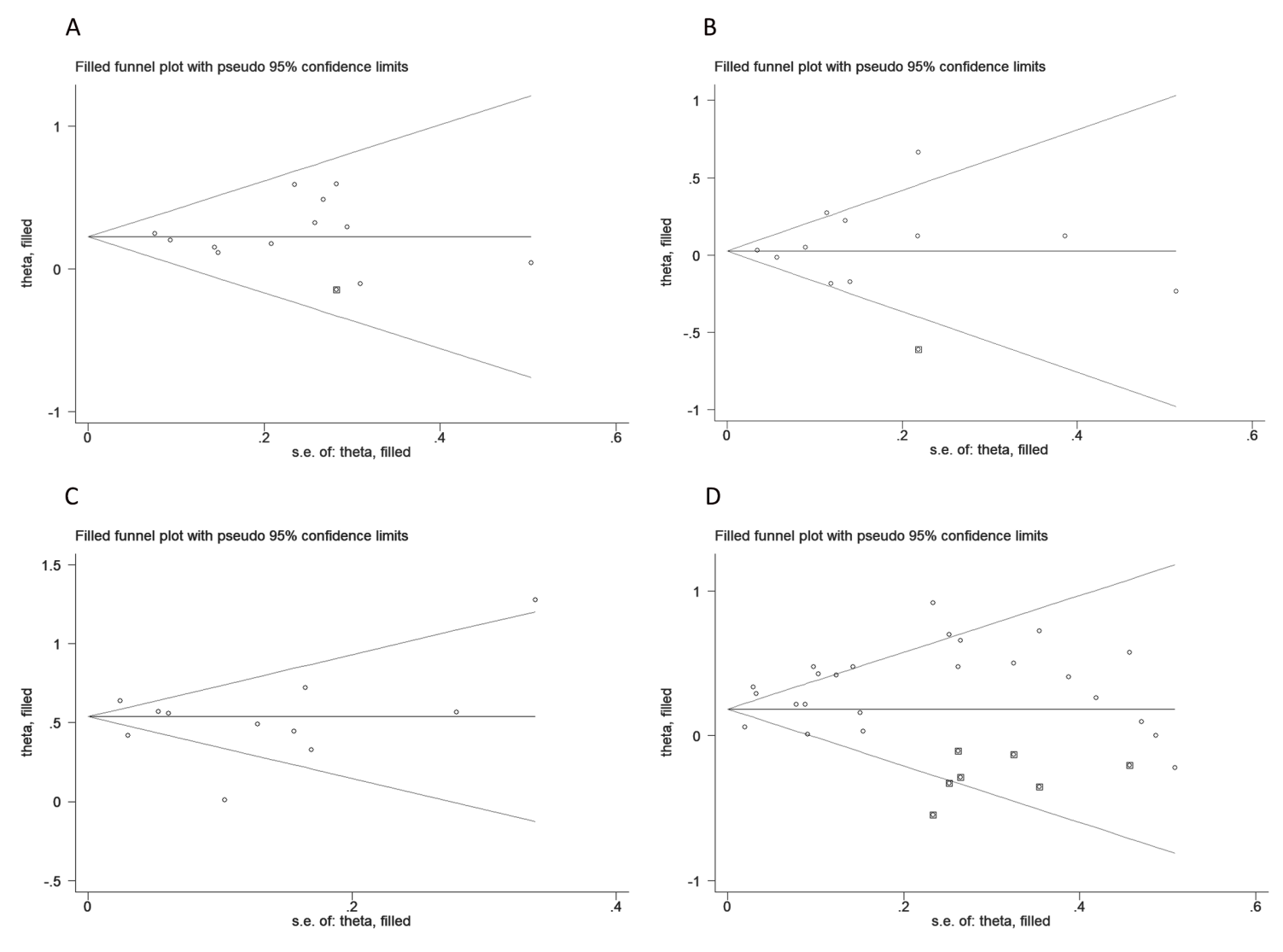


**Fig S7.** Sensitivity analysis on the association between overweight and digestive system cancers. (A) Pancreatic cancer; (B) Gastric cancer; (C) Liver cancer; (D) Colorectal cancer.


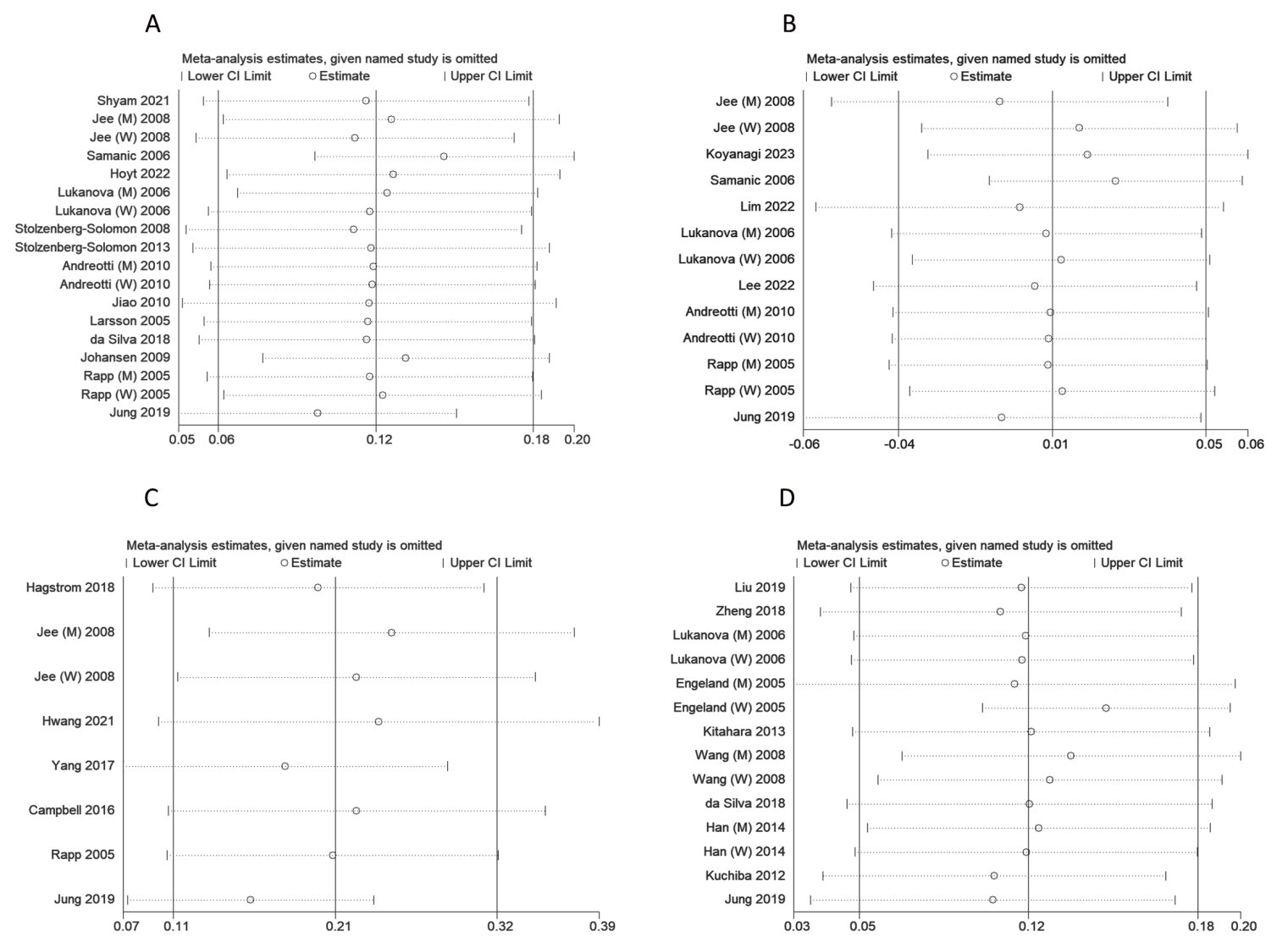


**Fig S8.** Sensitivity analysis on the association between obesity and digestive system cancers. (A) Pancreatic cancer; (B) Gastric cancer; (C) Liver cancer; (D) Colorectal cancer.


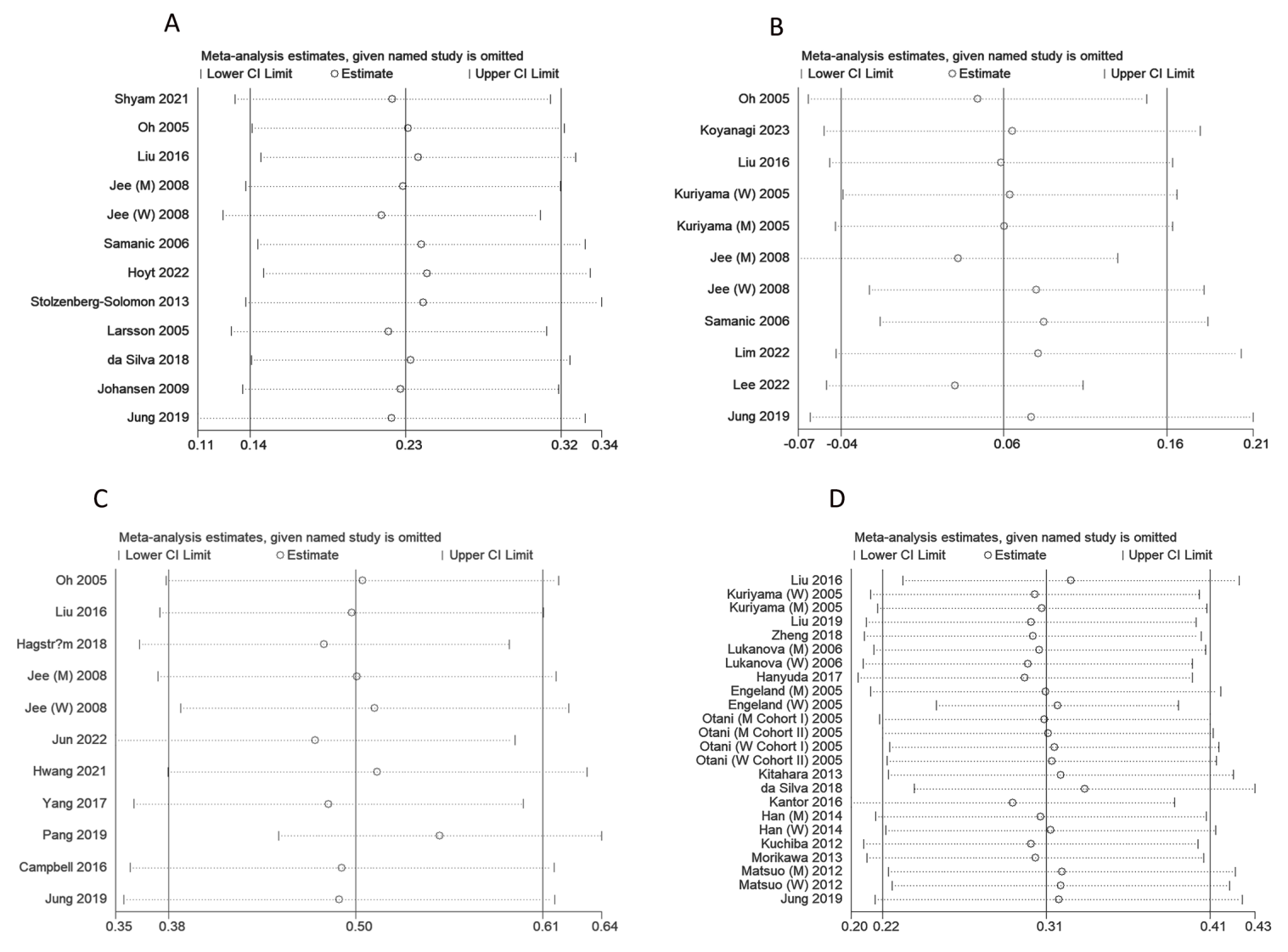

Supplement: S3 File — (DOCX) [file pone.0318256.s004.docx]
